# PARK7-Nrf2 axis mitigates NLRP3-driven inflammation to rescue neurovascular unit from hypoxia/reoxygenation-induced necroptosis

**DOI:** 10.1186/s12967-026-07898-5

**Published:** 2026-02-25

**Authors:** Zhiyuan Xie, Meifang Yang, Zhihan Liu, Zixuan Zhou, Zhiren Chen, Xia Zhang, Wenjuan Huang, Weiwei Chen, Shuqun Hu

**Affiliations:** 1 Department of Gastrointestinal Surgery, XuZhou Central Hospital/XuZhou Clinical School of Xuzhou Medical University/Xuzhou Central Hospital, Southeast University, Xuzhou, Jiangsu Province 221002 China; 2 Department of Neurology, XuZhou Central Hospital/XuZhou Clinical School of Xuzhou Medical University/Xuzhou Central Hospital, Southeast University/The Xuzhou School of Clinical Medicine of Nanjing Medical University, 199 Jiefang South Road, Xuzhou, Jiangsu Province 221000 China; 3 Gusu School of Nanjing Medical University, Suzhou, Jiangsu Province 215163 China; 4 Department of Neurology, The Affiliated Hospital of Xuzhou Medical University, Xuzhou, Jiangsu Province 221002 China; 5 Xuzhou Medical Science Research Institute, Xuzhou, Jiangsu 221009 China; 6 Laboratory of Emergency Medicine, Second Clinical Medical College of Xuzhou Medical University, Xuzhou, Jiangsu Province 221002 China; 7 Department of Emergency Medicine, The Affiliated Hospital of Xuzhou Medical University, Xuzhou, Jiangsu Province 221002 China; 8 Institute of Emergency Rescue Medicine, The Affiliated Hospital of Xuzhou Medical University, 99 Huaihai West Road, Xuzhou, Jiangsu China

**Keywords:** Acute ischemic stroke, Neurovascular unit, Necroptosis, NLRP3 inflammasome, PARK7, Nrf21

## Abstract

**Background:**

In the ischemic penumbra, necroptosis-induced inflammation exacerbates neurovascular unit (NVU) injury, though neuronal self-regulatory mechanisms are poorly understood. This study examined whether the PARK7–Nrf2 axis, a key antioxidant pathway, alleviates NLRP3-driven inflammation during necroptosis to protect the NVU.

**Methods:**

This study integrated a rat middle cerebral artery occlusion/reperfusion (MCAO/R) model in vivo and an in vitro model of primary cortical neuronal necroptosis induced by oxygen-glucose deprivation/reoxygenation (OGD/R) combined with the pan-caspase inhibitor Q-VD-Oph. Lentivirus-mediated gene knockdown and overexpression, pharmacological interventions including specific inhibitors (Nec-1), activators (tBHQ), and the protein translation inhibitor CHX were employed. These were combined with molecular biology techniques such as Western blot, immunofluorescence, co-immunoprecipitation, and RT-qPCR to systematically dissect the role and regulatory mechanisms of PARK7.

**Results:**

Hypoxia/reoxygenation injury robustly activated neuronal necroptosis, which subsequently triggered the assembly and activation of the NLRP3 inflammasome, leading to NVU disruption. Notably, PARK7 expression was significantly upregulated post-hypoxia/reoxygenation. Mechanistically, PARK7 directly enhanced Nrf2 activation through a dual mechanism: facilitating the dissociation of Nrf2 from Keap1 and promoting its nuclear translocation, while simultaneously stabilizing the Nrf2 protein and reducing its degradation. Activated Nrf2 subsequently upregulated the expression of downstream antioxidant proteins NQO1 and HO-1. This PARK7-Nrf2 signaling axis effectively suppressed the NLRP3 inflammasome and its associated inflammatory cascade. Consequently, PARK7 overexpression significantly alleviated NVU damage, reduced cerebral infarct volume, and improved neurological function, whereas PARK7 knockdown exacerbated these injuries. The detrimental effects caused by PARK7 deficiency were reversed by the Nrf2 activator tBHQ.

**Conclusion:**

This study reveals that PARK7 activates the Nrf2/ARE pathway, via promoting Nrf2-Keap1 dissociation and stabilizing Nrf2, to inhibit necroptosis-driven NLRP3 inflammasome overactivation in the ischemic penumbra. This mechanism provides a novel regulatory pathway for inflammation and highlights the PARK7/Nrf2 axis as a therapeutic target to mitigate neurovascular unit injury, thereby extending the treatment window and enabling a combined recanalization-cytoprotection strategy with significant translational promise.

## Introduction

**Fig. 1:**
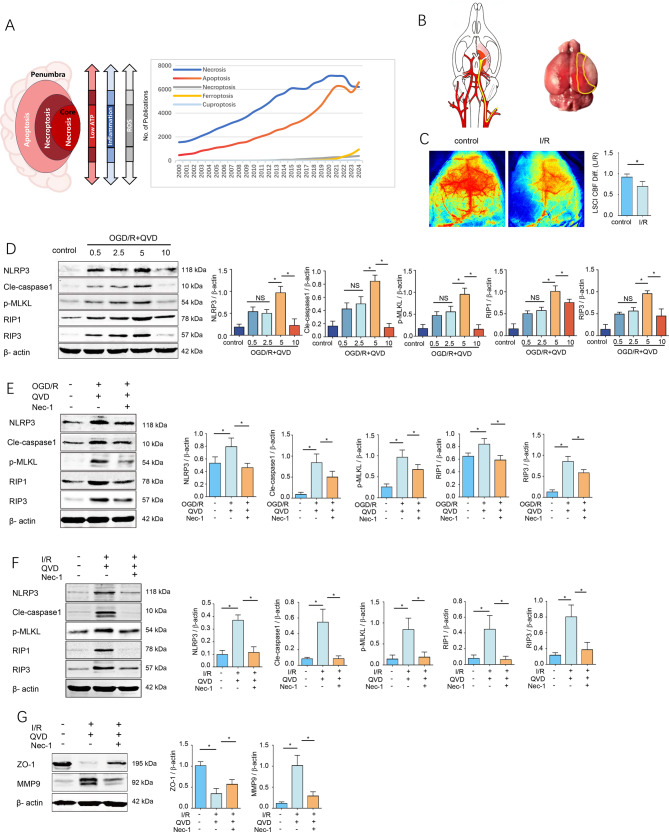
QVD exacerbates cerebral I/R-induced necroptosis in rats. (**A**) schematic representation of cell death modalities in the ischemic penumbra、trend analysis of publications on inflammation-associated cell death mechanisms (2020–2024); (**B**) Schematic diagram of the mouse MCAO model，the area within the yellow box represents the ischemic penumbra. (**C**) Cerebral blood flow measurement cortical cerebral blood flow was monitored by a laser speckle flow imaging technique; (**D**) in vitro: primary cortical neurons pretreated with QVD (0.5–10 μM) + OGD/R (2 h/12 h),Protein levels of NLRP3, RIP1, RIP3, p-MLKL, cleaved-caspase1 were detected by western blot, *n* = 4; (**E**) QVD (5 μM) + Nec-1 (20 μM) pretreatment 30 min,followed by OGD/R (2 h/12 h), NLRP3, RIP1, RIP3, p-MLKL and Cleaved-caspase1 were assessed by western blot, *n* = 4; (**F**) in vivo: MCAO (I/R 2 h/24 h) with QVD (1.5 mg/kg) ± Nec-1 (2 mg/kg),western blotting quantified protein expression of NLRP3, RIP1, RIP3,p-MLKL and cleaved caspase-1,*n* = 4; (**G**) Western blot analysis of ZO-1 and MMP-9 expression in brain tissues following cerebral I/R injury, *n* = 4.^*^p < 0.05

Cerebral infarction is an ischemic hypoxic pathological process caused by cerebral blood flow interruption or significant reduction, ultimately leading to neuronal death and severely impairing patients’ quality of life and prognosis [1]. The brain’s high metabolic demand and sensitivity to perfusion fluctuations make it particularly vulnerable to focal ischemic injury. During an acute ischemic stroke, a gradient of blood flow reduction extends from the relatively salvageable ischemic penumbra to the irreversibly damaged necrotic core, accompanied by progressive ATP depletion that exacerbates tissue damage [2] (Fig. 1A).

A critical step toward developing targeted stroke therapies lies in understanding the cell death pathways activated in the penumbra. Research over the past decade has established that the neurovascular unit (NVU) within this region suffers transient and reversible damage within hours post-stroke, defining a crucial therapeutic window for intervention [3, 4]. Our understanding of post-ischemic cell death has evolved beyond apoptosis to include regulated necrotic pathways such as necroptosis, pyroptosis, and ferroptosis. Of particular significance is necroptosis, a form of regulated necrosis that becomes the preferred death mechanism when energy levels are too low to execute apoptosis [2, 5]. This process is molecularly defined by the activation of the RIPK1–RIPK3-MLKL signaling axis. Its primary morphological features include organelle swelling and plasma membrane rupture, leading to the release of cellular contents and the initiation of robust inflammatory cascades [6]. This inflammation, often initiated by cytokines like tumor necrosis factor-α (TNF-α), can in turn stimulate further necroptosis in neighboring cells via the TNF/Fas pathway, creating a self-propagating cycle of cell death and inflammation [7].

Investigating the functional role of the NLRP3 inflammasome in neurons is crucial for a comprehensive understanding of the pathogenesis of central nervous system (CNS) inflammation. This research shifts the focus away from the traditional “glia-centric” paradigm and extends it to the neuron—the core functional unit of the nervous system [8]. While the role and activation of the NLRP3 inflammasome in microglia have been extensively studied, the regulatory mechanisms, activation pathways, and functional outcomes of neuronal NLRP3 remain a significant knowledge gap. Elucidating how neurons may actively engage in the initiation and amplification of inflammation via the NLRP3 inflammasome, rather than merely serving as passive recipients of injury, is central to uncovering common pathological mechanisms underlying neurodegenerative and cerebrovascular diseases [9, 10]. To address this, the present study utilizes in vitro primary cortical neuronal models to dissect these mechanisms, followed by in vivo validation at the neurovascular unit (NVU) level in a rat model. This integrated approach aims to establish a solid theoretical foundation for developing novel neuron-targeted anti-inflammatory strategies.

Cerebral Ischemia-Reperfusion Injury (CIRI) inherently represents a holistic impairment of the NVU. The efficacy of therapies targeting single neurons or isolated pathways is limited by this multifaceted pathology. Therefore, systematic intervention in the ischemic cascade is essential. Within this cascade, inflammation is a central hub, and necroptosis-driven inflammatory amplification is increasingly recognized as a pivotal mechanism in acute ischemic brain injury. This is especially relevant when pan-caspase inhibitors (e.g., zVAD, Q-VD-OPh) block apoptosis, leading to a significant reprogramming of cell death toward necroptosis [11, 12]. Consequently, targeting the inflammation induced by necroptosis has emerged as a promising strategy to extend the therapeutic time window. The NLRP3 inflammasome, a cytoplasmic multi-protein complex comprising NLRP3, ASC, and procaspase-1, serves as a critical inflammatory sensor. Upon activation, it cleaves procaspase-1 to its active form, catalyzing the maturation of pro-inflammatory cytokines IL-1β and IL-18, thereby amplifying the inflammatory cascade and exacerbating brain damage [13].

While the redox-sensitive protein PARK7 is known for its multifaceted antioxidant functions, its potential to regulate inflammatory cascades in acute cerebral infarction, particularly in the context of necroptosis, remains largely unexplored [14, 15]. This gap in knowledge highlights its potential as a novel therapeutic target. This study therefore employs Q-VD-OPh to induce necroptosis in rat cortical neurons in vitro and a middle cerebral artery occlusion/reperfusion (MCAO/R) model in vivo to investigate: 1) the role of the NLRP3 inflammasome in driving inflammation following necroptosis; 2) whether PARK7 can inhibit NLRP3 inflammasome activation by intervening in the necroptosis pathway; and 3) whether this mechanism alleviates neurovascular unit damage, thereby nominating PARK7 as an innovative therapeutic target for acute cerebral infarction.

## Methods

### Commercial sources of chemicals and reagents

Antibodies targeting caspase-1, ASC, Keap1, and Protein A-sepharose were sourced from Santa Cruz Biotechnology Inc. (Santa Cruz, CA, USA). Antibodies specific to NeuN, NLRP3, PARK7, NQO1, HO-1, RIP1, ZO-1, MMP9 and IL-1β were obtained from Abcam Inc. (Cambridge, MA, USA). Antibodies against RIP3 and Nrf2 were procured from Cell Signaling Technology Inc. (Danvers, MA, USA). The rat IL-1β quantikine ELISA kit was acquired from R&D Inc. (Minneapolis, MN, USA). Q-VD-OPh (QVD), tert-butylhydroquinone (tBHQ), cycloheximide (CHX), dihydroethidium (DHE), poly-D-lysine hydrobromide, glutamine, dimethyl sulfoxide, and penicillin were purchased from Sigma-Aldrich Inc. (St. Louis, MO, USA). Dulbecco’s modified Eagle medium (DMEM), trypsin-EDTA, HEPES, B27 supplement, and β-mercaptoethanol were supplied by Gibco Inc. (Rockville, MD, USA). The RNA PCR Kit and RNAiso™ Plus were provided by Takara Biotechnology Inc. (Dalian, Liaoning, China).

### Animals

Pregnant Sprague-Dawley rats (*n* = 36, gestational age 18–21 days) and adult male Sprague-Dawley (SD) rats (*n* = 235) were obtained from the Xuzhou Medical University Laboratory Animal Center. All experimental protocols adhered to the rules established by the Animal Care and Use Committee of Xuzhou Medical University (Ethics Approval Number: 202212S007).

### Primary cortical neuron culture

Culture dishes were pre-treated with poly-L-lysine (0.1 mg/mL) 24 hours before utilisation. Prior to commencing the experiment, the plates were meticulously cleansed with sterilised MilliQ water. Pregnant SD rats (gestational age 18–21 days) were euthanised under ether anaesthesia, and foetal brains were swiftly excised. The meninges and blood arteries were meticulously excised, and the cortical tissues were separated and stored on ice in h-DMEM (supplemented with 1% penicillin-streptomycin). The cortical tissues were rinsed, chopped into approximately 1 mm^2^ pieces, and centrifuged to eliminate the supernatant. The tissue pellet was further digested with 0.25% trypsin-EDTA at 37 °C for 10 to 15 minutes. Trypsin was neutralised by the addition of foetal bovine serum, succeeded by three washes with DMEM. Cells were resuspended in serum-free neurobasal media, which included B27 supplement at a concentration of 2 mL per 100 mL and 0.5 mM glutamine. The suspension was filtered through a 70 μm mesh and quantified. Neurons were grown at a density of 1 × 10^5^ cells/cm^2^ in poly-L-lysine-coated dishes and incubated at 37 °C in 5% CO₂. The medium was partially replenished 2 to 3 times weekly, and cell maturation was observed till day 12. Neuronal purity was verified by immunostaining for NeuN and DAPI.

### Oxygen-glucose deprivation (OGD) protocol

Following 12 days in vitro, primary cortical neurones were rinsed with pre-warmed glucose-free DMEM and subsequently incubated in glucose-free DMEM augmented with QVD (5 μM) at 37 °C for 30 minutes. Cultures were subsequently positioned in a hypoxic chamber with oxygen levels decreased to less than 1.5% for a duration of 2 hours. Subsequent to OGD, the neurones were reintroduced to a glucose-enriched growth medium (supplemented with 5 μM QVD), and the cultures were cultured under normoxic conditions at 37 °C for additional tests.

### Middle cerebral artery occlusion (MCAO) model

Adult male SD rats (200-250 g) were anesthetized with 1.5% pentobarbital sodium via intraperitoneal injection. A midline incision in the neck was performed to expose the right common carotid artery (CCA), external carotid artery (ECA), and internal carotid artery (ICA). The ECA was ligated, and a nylon monofilament was inserted into the ICA to induce middle cerebral artery occlusion (complete cessation of blood flow). The suture was advanced approximately 18 ± 1 mm to achieve occlusion. After 2 hours of ischemia, reperfusion was induced by withdrawing the filament (Fig. 1B). Sham-operated rats underwent identical procedures, excluding occlusion. Q-VD-OPH (1.5 mg/kg) was injected intraperitoneally 30 minutes prior to ischemia and at the onset of reperfusion to suppress apoptosis. Brain tissue from the ischemic penumbra was collected for further analysis (The parietal cortex was collected from the peripheral regions of macroscopically visible necrotic areas as shown in Fig. 1B).

### Neurological scoring and inclusion/exclusion criteria

Neurological deficits were evaluated post-recovery utilising a 5-point scoring system derived from the methodologies of Longa and Bederson. 0: No neurological deficits; 1: Incomplete extension of the opposite forelimb; 2: Turning towards the opposite side; 3: Falling towards the opposite side; 4: Inability to walk or loss of consciousness. Subjects scoring between 1 and 3 were incorporated into the study. Animals scoring < 1 or >4, as well as those that succumbed due to anaesthesia, surgical complications, or subarachnoid haemorrhage, were excluded.

### Brain water content quantification

After 24 h of reperfusion, rats were decapitated and brains were rapidly harvested. Whole brains were immediately weighed to obtain wet weight (recorded to 0.1 mg precision) and placed in a 100 °C oven for 24 h. The dried brain tissues were then re-weighed to determine dry weight. Brain water content (%) was calculated as [(wet weight - dry weight)/wet weight] × 100%.

### Western blot analysis

Protein concentrations were quantitatively assessed using the BCA assay, with BSA serving as the standard reference. Subsequently, 100 micrograms of protein samples were fractionated by SDS-PAGE and transferred onto nitrocellulose membranes. These membranes were then immunoblotted with a panel of primary antibodies specifically targeting NLRP3, ASC, IL-1β, caspase-1, caspase-3, RIP1, RIP3, MLKL, PARK7, NQO1, HO-1, Nrf2, Keap1, ZO-1, MMP9 and β-actin. Following incubation with fluorescent secondary antibodies, the membranes were visualized using an Odyssey infrared imaging system to detect and quantify the corresponding protein signals.

### LDH release assay

Lactate dehydrogenase (LDH) levels in the culture supernatants were assessed with an automated biochemical analyzer.

### Immunofluorescence

After anesthesia, the rats were subjected to cardiac perfusion with 4% paraformaldehyde (PFA). The brains were then extracted and fixed in 4% PFA at 4 °C for 24 hours. Following fixation, the brains were sectioned into 10 μm-thick frozen slices and stored at −20 °C for subsequent experiments. For the immunostaining procedure, the frozen sections or isolated cells were fixed again with 4% PFA, followed by immunostaining using specific antibodies targeting NLRP3 and NeuN. Nuclei were counterstained with DAPI. Finally, the samples were imaged and analyzed using a confocal laser scanning microscope (CLSM, Leica STELLARIS 5, Germany).

### Enzyme-linked immunosorbent assay (ELISA)

The concentration of IL-1β in the culture supernatant was quantified using an ELISA, following the manufacturer’s protocol of a commercially available ELISA kit. Absorbance was measured at a wavelength of 450 nm, and the cytokine concentration was determined by interpolation from a standard curve.

### Isolation of nuclear proteins

Nuclear and cytoplasmic proteins were extracted using a Nuclear Protein Extraction Kit (Beyotime) following the manufacturer’s instructions. Initially, 200 μL of cytoplasmic extraction buffer A (containing PMSF) was mixed with 20 μL of cell pellet. The mixture was briefly vortexed for 5 seconds, kept on ice for 15 minutes, and then 10 μL of cytoplasmic extraction buffer B was introduced. After another 5-second vortex, the sample was incubated for 1 minute, vortexed once more, and centrifuged at 15,000 g for 5 minutes at 4 °C. The resulting supernatant, which contained the cytoplasmic proteins, was collected into pre-chilled tubes and stored at −80 °C. For the isolation of nuclear proteins, the remaining pellet was mixed with 50 μL of nuclear extraction buffer (supplemented with PMSF) and vortexed for 30 seconds. This mixture was then kept on ice and vortexed briefly every 1–2 minutes over a 30-minute period. After a final centrifugation at 15,000 g for 10 minutes at 4 °C, the supernatant containing the nuclear proteins was collected and stored at −80 °C for further analysis.

### Lentivirus-mediated RNA interference

Primary cortical neurons were cultivated from pregnant rats for a duration of five days. After verifying robust cell proliferation under a microscope, the cells were subjected to lentiviral infection for a duration of 12 hours. Subsequent to infection, the cells were rinsed thrice with pre-warmed DMEM at 37 °C and subsequently cultured in neuronal growth medium (neurobasal media, 100 mL, augmented with 2 mL B27 and 0.5 mmol/L glutamine) within a 37 °C incubator containing 5% CO₂ for an additional 96 hours. The transfection efficiency was evaluated using a fluorescent microscope, achieving a transfection rate greater than 85%. Adult rats were anaesthetised with 10% chloral hydrate (3.5 mL/100 g, administered intraperitoneally). The lateral ventricle was targeted using stereotaxic coordinates: 0.8 mm posterior to the bregma, 1.5 mm lateral to the midline, and 4.5 mm in depth. A little cranial aperture was created at the designated location, and 6 μL of lentivirus was administered into the right lateral ventricle at a velocity of 300 nL/min utilising a microinjection pump. Following the injection, the needle remained in situ for 10 minutes prior to gradual removal. The incision was sutured, cleansed with iodine, and the rats were permitted to recuperate in their cages, and continue feeding for 5 days. The PARK7 siRNA target sequence was: 5’-GGGUGCACAGAACUUAUCUTT-3’.

### Total RNA was reverse transcribed

Isolation of total RNA from rat Primary Cortical Neurons and brain tissue was accomplished with TRIzol reagent based on specifications of the manufacturer. Total RNA was converted to cDNA using the Takara PrimeScript™ RT Reagent Kit following the manufacturer’s protocol. The reverse transcription reaction was assembled on ice, with conditions set to 37 °C for 15 minutes for cDNA synthesis, followed by heat inactivation at 85 °C for 5 seconds and cooling to 4 °C. PCR amplification was performed using the LightCycler 480 SYBR Green 1 master mix. The reaction system was prepared following the manufacturer’s guidelines and loaded into a real-time quantitative PCR instrument. The amplification was performed using the LightCycler/LightCycler 480 System protocols. Following the completion of the reaction, the amplification and melt curves were analyzed. GAPDH served as the internal control, and the relative expression of PARK7 and Nrf2 was determined using the 2-^(ΔΔCt)^ method. The primer sequences were as follows: PARK7 forward primer 5′-TGGCTCACGAAGTAGGCTTT-3’ and reverse primer 5′- AGGGCTTGGGCTCTCTAGTC-3′; Nrf2 forward primer 5′-ATCCAGACAGACACCAGTGGATG-3′ and reverse primer 5′- GGCAGTGAAGACCGAACTTTCA-3′; GAPDH forward primer 5′-GATGCTGGTGCTGAGTATGTCG-3′ and reverse primer 5′-GTGG TGCAGGATGCATTGCTGACA-3′.

### Superoxide anion fluorescent probe (DHE) assay

The quantification of superoxide anions was performed through DHE fluorescence staining. A 10 µM stock solution, maintained at −20 °C, was diluted in culture medium to achieve a 1.0 µM working concentration. Cellular loading was accomplished by 30-minute incubation at physiological temperature (37°C), followed by triple washing with pre-warmed DMEM to ensure complete removal of unincorporated dye. Fluorescence intensity was subsequently quantified using confocal microscopy.

### Immunoprecipitation

For immunoprecipitation, protein samples (400 μg) were dissolved in immunoprecipitation buffer (IP buffer) and combined with 2 μg of primary antibody. The total volume was adjusted to 500 μL with IP buffer. The mixture was incubated overnight at 4 °C on a rotator. The following day, 20 μL of Protein A-Sepharose was added and gently shaken for 2 hours at 4 °C. After centrifugation at 10,000 g for 2 minutes, the supernatant was discarded. The pellet was washed three times with 600 μL of IP buffer, with centrifugation at 10,000 g for 2 minutes after each wash. Finally, the pellet was resuspended in 25 μL of IP buffer and loading buffer, boiled for 5 minutes, and centrifuged to obtain the supernatant. The immunoprecipitated protein samples were then analyzed via Western blot.

### Measurement of cerebral infarction volume by TTC staining

After euthanasia, brains were carefully harvested and immediately frozen at −20 °C for 20 min. Coronal sections (2-mm thick) were prepared using a brain matrix. Sections were immersed in 2% 2,3,5-triphenyltetrazolium chloride (TTC) solution and incubated at 37 °C for 15–30 min in the dark with gentle agitation. Viable brain tissue was stained red due to formazan formation, while infarcted areas remained unstained. After staining, sections were fixed in 4% paraformaldehyde for 4 h and photographed. Infarct volume was quantified using Image Pro Plus 6.0 software. The percentage of infarct volume was calculated as follows: (infarct volume/total brain volume) × 100%.

### Statistical analysis

All statistical evaluations were carried out utilizing SPSS version 25.0 (IBM Corporation, Armonk, NY, USA). Quantitative variables were presented as means accompanied by their standard deviations. Inter-group differences were assessed through Student’s t-test for dual-group comparisons, whereas multiple group analyses employed the ANOVA method. *p* < 0.05 was considered statistically significant.

## Results

### Ischemia-reperfusion injury induces necroptosis in the rat neurovascular unit

Neuroinflammation has become a central focus in the pathological research of acute cerebral infarction. Beyond traditional necrosis and apoptosis pathways, necroptosis emerges as the predominant cell death modality in energy-compromised cerebral lesion areas. Research on its associated inflammatory cascades has been progressively increasing annually (Fig. 1A). In this study, we employed a middle cerebral artery occlusion (MCAO) mouse model to simulate ischemic conditions (Fig. 1B). Cerebral blood flow was assessed using laser speckle flow imaging, which further revealed exacerbated ischemic cerebral tissue damage in the MCAO model (Fig. 1C). QVD is an irreversible pan-caspase inhibitor that has been shown to inhibit apoptosis more effectively than the commonly used inhibitors zVAD-fmk and Boc-D-fmk [16–18]. QVD suppresses apoptosis mediated by the three major apoptotic pathways—caspase 9/3, caspase 8/10, and caspase 12, while also exerting a weaker inhibitory effect on caspase1 [18]. Our in vitro experiments demonstrated that after treatment with varying concentrations of QVD (0.5 μM, 2.5 μM, 5 μM, 10 μM), followed by 2 hours of hypoxia and 12 hours of reoxygenation, resulted in an elevation of NLRP3 and Cle-caspase1 expression levels, with a maximum observed at 5 μM QVD. At a concentration of 10 μM, there was a suppression in the expression of Cle-caspase1, accompanied by a decrease in NLRP3 levels, with these changes being statistically significant. The expression of necroptosis markers RIP1, RIP3, and p-MLKL also peaked at 5 μM, prompting the selection of this concentration for further investigations (Fig. 1D).

Nec-1, a specific necroptosis inhibitor, was utilized to investigate the occurrence of necroptosis induced by OGD/R+QVD. In the cellular experiments, the addition of Nec-1 resulted in a significant reduction in the protein levels of NLRP3, Cle-caspase1, and RIP1, RIP3, and p-MLKL, compared to the OGD+QVD group (Fig. 1E). Administration of QVD-OPH induced necroptosis in rat MCAO models, while treatment with Necrostatin-1 (Nec-1) markedly suppressed this process. The inhibition of necroptosis significantly reduced NLRP3 inflammasome levels and the expression of necroptotic markers (RIP1, RIP3, and p-MLKL) compared to the I/R+QVD-OPH control group (Fig. 1F). Additionally, I/R injury resulted in significant blood-brain barrier disruption. In the QVD-OPH-treated group, a marked increase in matrix metalloproteinase-9 (MMP-9) expression was observed, accompanied by a significant decrease in zonula occludens-1 (ZO-1) protein levels relative to control groups (Fig. 1G). These findings, observed at cellular and in vivo levels, confirm that necroptosis mediates ischemic damage to the neurovascular unit (NVU), particularly involving cortical neuronal death under hypoxia-ischemia conditions.

### NLRP3 inflammasome activation exacerbates neurovascular unit injury via promoting necroptosis in ischemic stroke rats

**Fig. 2:**
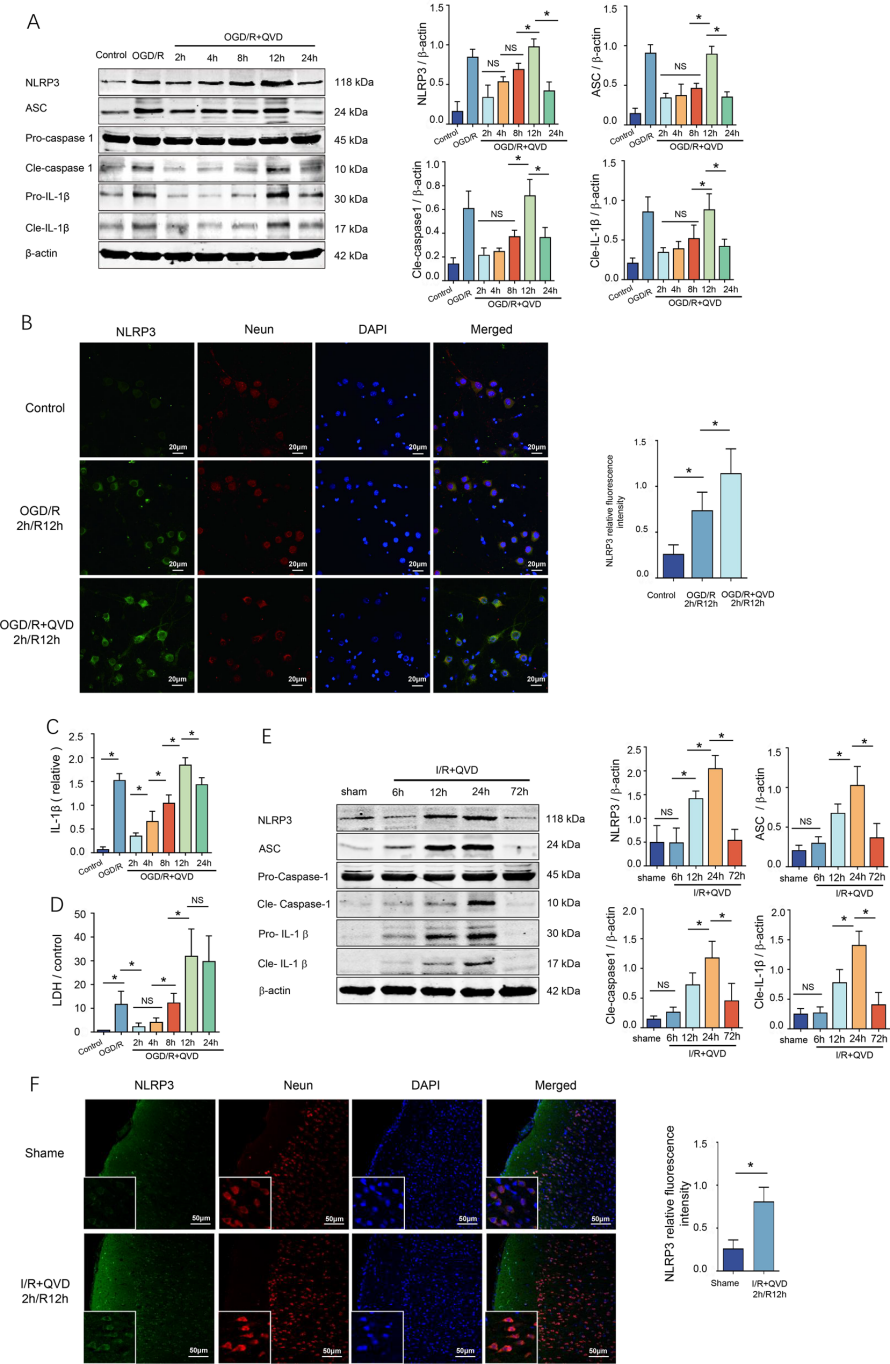
Expression of the NLRP3 inflammasome in the necroptosis pathway induced by cerebral ischemia-reperfusion. (**A**) Primary cortical neurons were cultured for 12 days and pretreated with QVD (5 μM) for 30 minutes, followed by 2 hours of hypoxia and reoxygenation at different time points (2 h, 4 h, 8 h, 12 h, 24 h). Cells in the control and OGD groups were collected after 12 hours of reoxygenation. The expression levels of NLRP3 inflammasome components were analyzed by Western blot, *n* = 4; (**B**) NLRP3 immunofluorescence in cortical neurons after 12-h reoxygenation (confocal microscopy, 400×), *n* = 4; (**C**) the levels of IL-1β in the culture medium of each group were detected by ELISA, *n* = 4; (**D**) Post-reoxygenation supernatants were analyzed for LDH,*n* = 6; (**E**) Rats received QVD (1.5 mg/kg, i.P.) 30 min pre-ischemia and pre-reperfusion. NLRP3 inflammasome was quantified by WB after 2 h ischemia/6-72 h reperfusion,*n* = 4; (**F**) Double immunofluorescence staining with anti-NLRP3 and anti-neun in cortical neurons, *n* = 6. ^*^p < 0.05

The NLRP3 inflammasome is primarily expressed in inflammatory cells, including astrocytes, microglia, and macrophages, whereas its expression levels in neurons are comparatively lower. Due to this, research on inflammatory mediators is less frequently conducted in primary cortical neurons [19]. The objective of this study is to elucidate the involvement of the NLRP3 inflammasome in neuronal necroptosis. At the cellular level, necroptosis was induced through the application of OGD/R + QVD. The results demonstrated that the expression levels of NLRP3 inflammasome components, including NLRP3, ASC, and pro-caspase-1, progressively increased with prolonged reoxygenation time, reaching their peak at 12 hours post-reoxygenation. (Fig. 2AB). Concurrently, western blot analysis showed elevated levels of the activated proteins Cle-caspase1 and Cle-IL-1β, with ELISA results also indicating a peak in Cle-IL-1β expression in the cell culture medium at 12 hours of reoxygenation (Fig. 2AC). The extent of neuronal damage, as measured by LDH, also peaked at 12 hours of reoxygenation, positively correlating with the activation of the NLRP3 inflammasome (Fig. 2D). Immunofluorescence analysis demonstrated a marked upregulation of NLRP3 expression in both the OGD and OGD+QVD groups relative to the normal group, aligning with the western blot data, and the protein was predominantly localized within the cytoplasmic compartment (Fig. 2B).

In the rat MCAO model, necroptosis was triggered, and the expression of NLRP3 inflammasome components progressively increased with prolonged reperfusion, reaching a peak at 24 hours. Similarly, the levels of activated Cle-caspase1 and Cle-IL-1β also peaked at 24 hours (Fig. 2E). Using anti-Neun for immunofluorescence staining to label neurons, the results showed that NLRP3 expression in the QVD group was notably higher compared to the normal control group, with NLRP3 specifically localized within the cytoplasm (Fig. 2F).

### tBHQ attenuates necroptosis and protects rat cortical neurons via promoting Nrf2 nuclear translocation

**Fig. 3:**
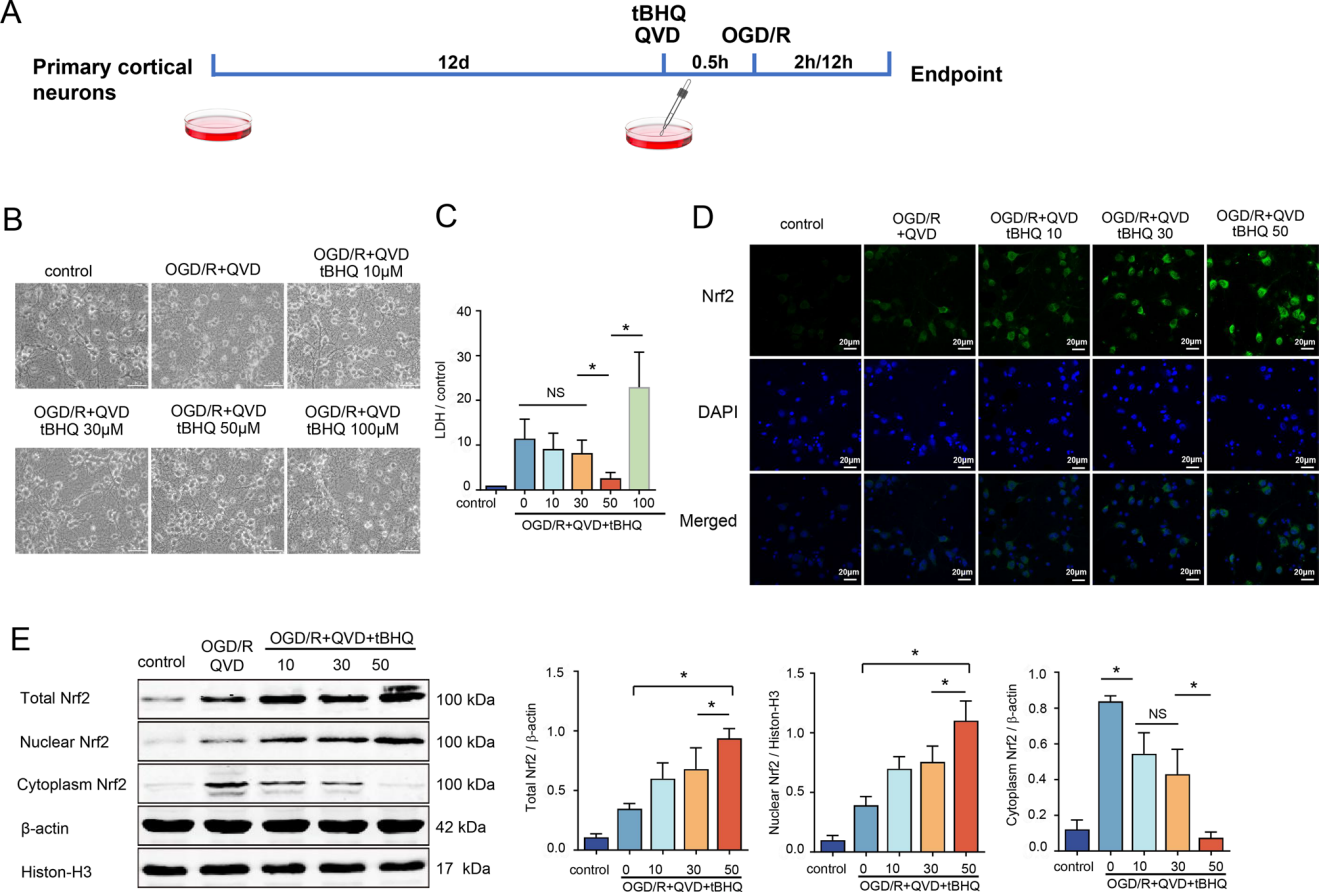
tBHQ promotes Nrf2 nuclear translocation to safeguard rat cortical neurons. Primary cortical neurons were cultured for 12 days, pretreated with QVD (5 μM), tBHQ (10, 30, 50, 100 μM) for 30 min, then subjected to 2 h oxygen-glucose deprivation (OGD)/12 h reoxygenation, *n* = 4; (**A**) Schematic diagram of necroptosis induced by OGD/R in primary cortical neurons treated with tBHQ, *n* = 4; (**B**) Cell morphological alterations were examined microscopically at 200 x magnification, *n* = 6; (**C**) LDH concentrations in the cell culture media were quantified, *n* = 6; (**D**) Immunofluorescence analysis of Nrf2 nuclear translocation in neurons subjected to varying doses of tBHQ (10 μM, 30 μM, 50 μM) within the necroptosis signaling pathway, with nuclei labeled with DAPI, *n* = 6; (**E**) Western blot analysis was employed to assess Nrf2 protein levels in total protein, nuclear fractions, and cytoplasmic fractions after pretreatment with varying dosages of tBHQ, *n* = 4. ^*^p < 0.05, for indicated comparisons

Tert-butylhydroquinone (tBHQ) is a synthetic polyphenolic antioxidant and a potent inducer of antioxidant proteins, with various cytoprotective effects. It stimulates the Nrf2 protein, facilitating its nuclear translocation, augmenting its interaction with the antioxidant response element (ARE), and modulating the expression of genes encoding antioxidant proteins [20]. The experimental protocol for tBHQ-mediated regulation of OGD/R-induced necroptosis in primary cortical neurons is illustrated (Fig. 3A). Microscopic examination of the OGD/R + QVD group showed significant neuronal swelling, deformation, and vacuolization (Fig. 3B). When tBHQ was administered at different concentrations (10 μM, 30 μM, 50 μM, 100 μM), the results indicated that as tBHQ concentration increased, LDH levels progressively decreased, reaching the lowest point at 50 μM, with a significant difference compared to the OGD/R + QVD group (Fig. 3C). At this concentration, neuronal swelling was notably reduced under the microscope, and most cells maintained their original morphology with clear halos around them (Fig. 3B). However, when the concentration of tBHQ was further increased to 100 μM, LDH levels rose, and microscopy revealed increased cellular swelling, intracellular vacuoles and granules, axonal breakage, and the presence of fragmented debris, indicating increased cell damage (Fig. 3BC). Therefore, three lower concentrations of tBHQ (10 μM, 30 μM, 50 μM) were selected for the following study. Immunofluorescence analysis revealed that the nuclear translocation of Nrf2 in neurons increased correspondingly with the elevation of tBHQ concentration (Fig. 3D). Western blot analysis was conducted for total Nrf2, cytosolic, and nuclear proteins. The results indicated that at a concentration of 50 μM, both total Nrf2 and nuclear Nrf2 protein levels reached their peaks. (Fig. 3E), suggesting that in the mechanism of Necroptosis, tBHQ activates Nrf2, promotes its nuclear translocation, and protects neurons. Hence, 50 μM tBHQ was selected for subsequent experiments.

### PARK7 confers protection against cerebral ischemia-reperfusion injury by suppressing neuronal necroptosis via Nrf2 activation

**Fig. 4:**
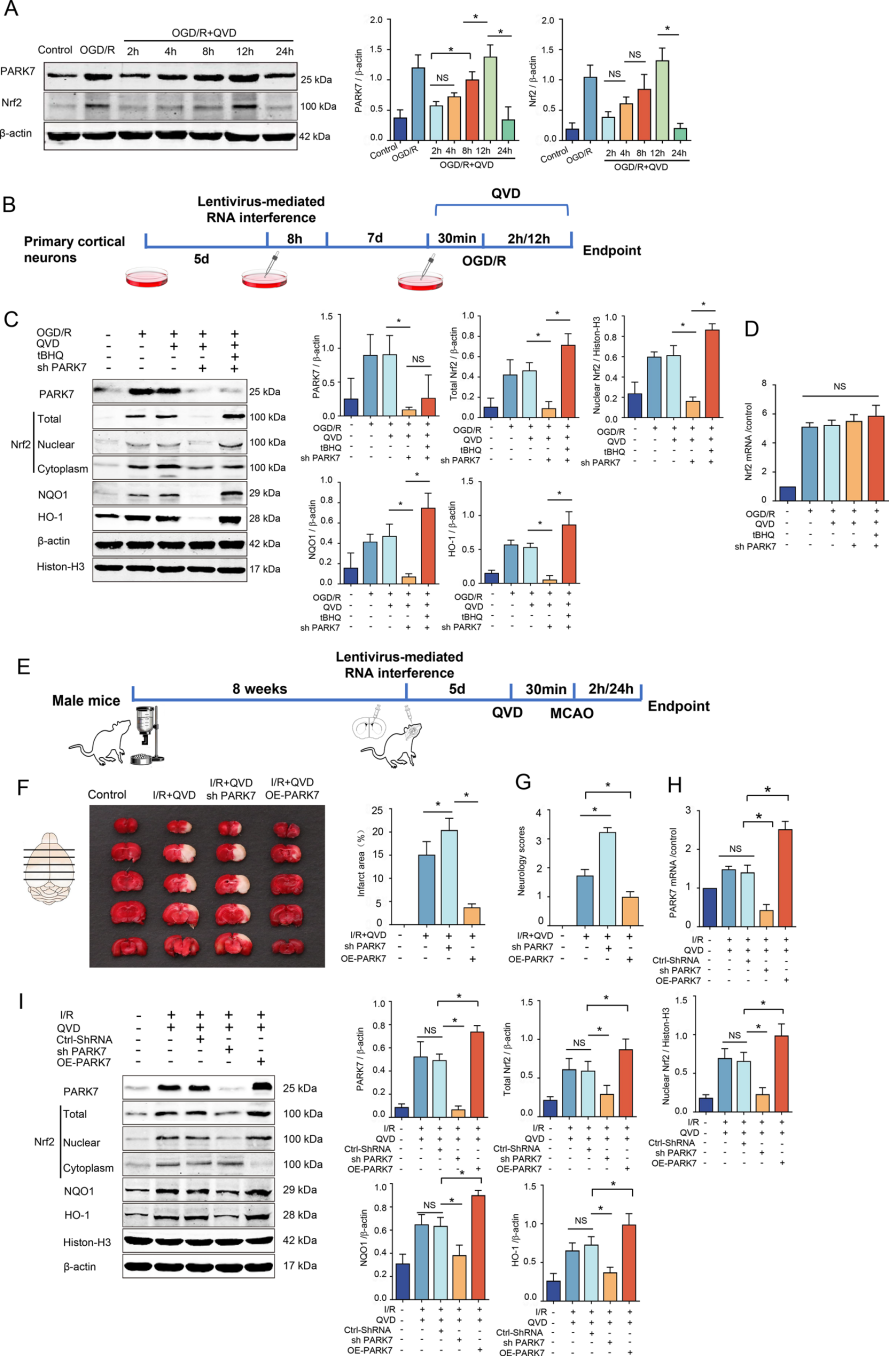
PARK7 offers neuroprotection against necroptosis in ischemia-hypoxia- challenged rat cortical neurons through Nrf2 signaling. (**A**) After 12 days in culture, primary cortical neurons were pretreated with QVD (5 μM, 30 min), subjected to OGD for 2 h, and then reoxygenated for varying durations (2, 4, 8, 12, 24 h). PARK7 and Nrf2 protein levels were assessed by Western blot, *n* = 4; (**B**) Schematic of rat cortical neuron experiments with adenoviral interference: lentivirus was added on day 5, medium replaced after 8 h, and continued until day 12. Cells were then pretreated with QVD (5 μM) and tBHQ (50 μM) for 30 min, followed by OGD 2 h and reoxygenation 12 h; (**C**) Western blot analysis detected PARK7, Nrf2 (total, nuclear, cytoplasmic), NQO1, and HO-1 protein levels. β-actin and histone-H3 served as internal controls for cytoplasmic/total and nuclear proteins, respectively, *n* = 4; (**D**) RT-qPCR was used to detect Nrf2 RNA levels, and *n* = 4; (**E**) Experimental flowchart for the rat MCAO model: following lentiviral interference, rats underwent 2 h ischemia and 24 h reperfusion, with QVD (1.5 mg/kg, i.P.) administered 30 min pre-ischemia and at reperfusion onset.; (**F**) TTC staining of rat brain tissue was performed, *n* = 4; (**G**) Neurological function scores for each group were analyzed, *n* = 4; (**H**) RT-qPCR detected PARK7 RNA expression, *n* = 4; (**I**) in vivo mouse study: Western blot analysis was performed to detect protein levels of PARK7, Nrf2 (in total, nuclear, and cytoplasmic fractions), NQO1 and HO-1. β-actin and histone-H3 served as loading controls for cytoplasmic/total and nuclear proteins, respectively, *n* = 4. ^*^p < 0.05

PARK7 and Nrf2 are both proteins associated with the antioxidant stress response. Western blot analysis revealed that with the prolonged reoxygenation time following OGD/R+QVD-induced cortical neuron injury in rats, the expression levels of PARK7 and Nrf2 proteins showed an increasing trend, peaking at 12 hours of reoxygenation (Fig. 4A). To investigate the regulation of PARK7 on Nrf2 and its downstream antioxidant proteins in the necroptosis signaling pathway following ischemic reperfusion injury, lentiviral-mediated knockdown of PARK7 was conducted (Fig. 4B). Western blot analysis revealed that, compared to the OGD+QVD group, both the total Nrf2 protein levels and nuclear protein levels were significantly reduced in the PARK7 knockdown group, accompanied by a corresponding downregulation of Nrf2-regulated transcriptional proteins NQO1 and HO-1 in the nucleus (Fig. 4C). RT-qPCR results showed no significant difference in Nrf2 mRNA levels among the experimental groups (Fig. 4D). Upon addition of tBHQ to the knockdown group, Nrf2 nuclear translocation increased again, and both NQO1 and HO-1 proteins were upregulated (Fig. 4C). These experiments demonstrated that tBHQ could promote the increase of Nrf2 protein levels and enhance nuclear translocation in PARK7-knockdown cells.

PARK7 exerts cytoprotective effects by regulating antioxidant proteins.The experimental flowchart for the rat MCAO model is shown in Fig. 4E: after lentiviral interference, focal cerebral ischemia was induced for 2 h followed by 24 h of reperfusion, and QVD (1.5 mg/kg, i.p.) was administered 30 min before ischemia and at the onset of reperfusion. In vivo rat studies, following lentiviral interference with PARK7 and TTC staining to assess ischemic necrosis in the brain after middle cerebral artery occlusion, it was found that the ischemic necrosis area was increased in the I/R+QVD+Sh PARK7 group compared to the control group, while it was decreased in the I/R+QVD+OE-PARK7 group (Fig. 4F). A comparison of neurological function scores across the groups revealed that the PARK7-knockdown group had higher scores than the control group, whereas the PARK7-overexpression group exhibited significantly lower scores, with these differences being statistically significant (Fig. 4G). Following adenovirus-mediated interference with PARK7 expression, RT-PCR assessment showed significantly reduced PARK7 mRNA levels in the I/R+QVD+Sh PARK7 group versus controls, whereas the I/R+QVD+OE-PARK7 group exhibited significantly elevated levels compared to controls (Fig. 4H). Western blot analysis of Nrf2 nuclear translocation and activation revealed that nuclear Nrf2 protein levels decreased after PARK7 knockdown, accompanied by decreased levels of its downstream transcriptional proteins HO-1 and NQO1. Conversely, nuclear Nrf2 protein levels increased after PARK7 overexpression, with synchronous increases in HO-1 and NQO1 levels (Fig. 4I). In summary, PARK7 regulates oxidative stress responses in the necroptosis signaling pathway by acting on the downstream Nrf2/ARE pathway. We infer that the target of PARK7 is Nrf2, the same as that of tBHQ.

### The negative regulatory effect of PARK7 on NLRP3 inflammasome in necroptosis of rat cortical neurons induced by ischemia and hypoxia can Be reversed by tBHQ

**Fig. 5:**
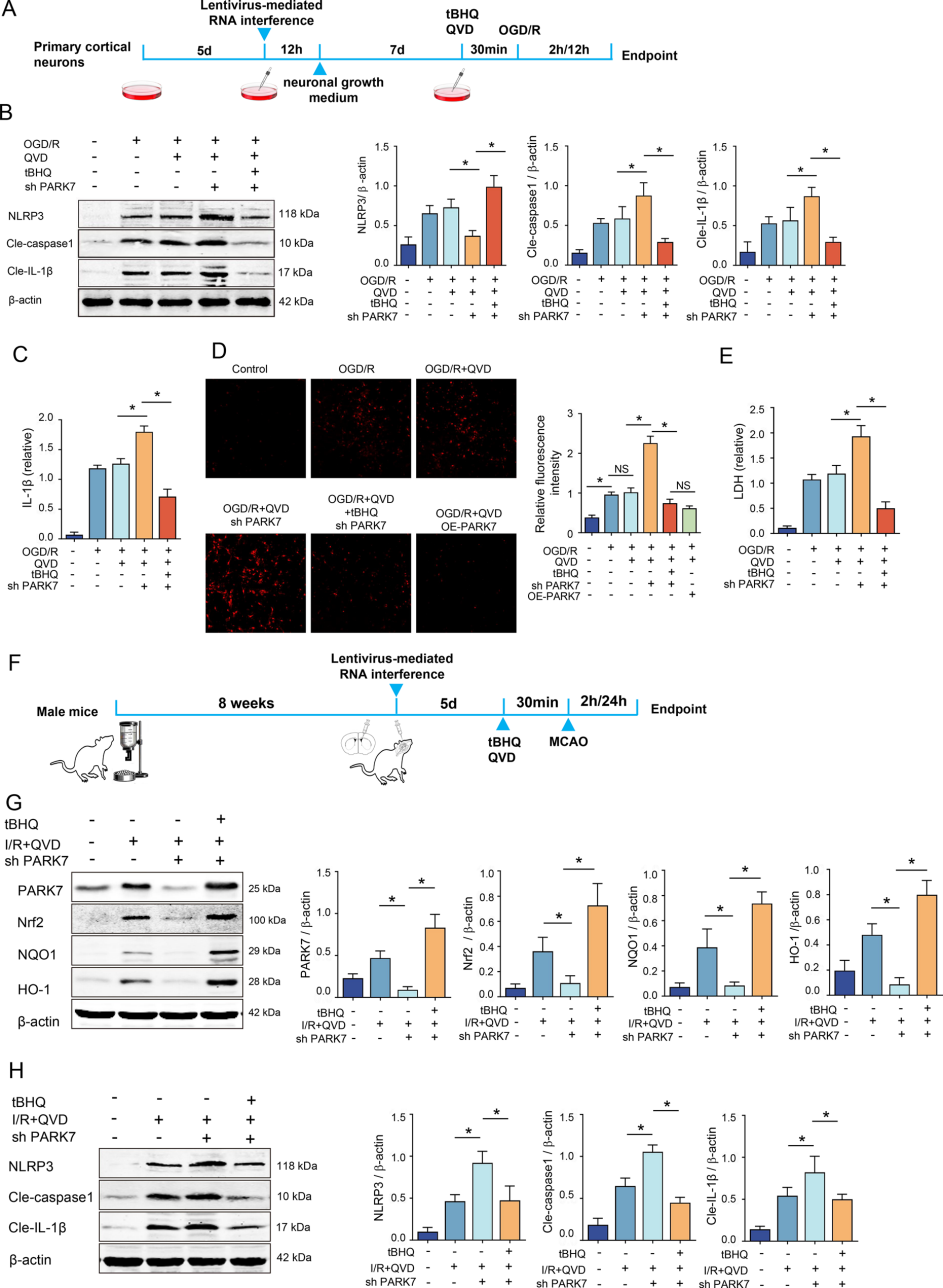
tBHQ reverses PARK7‘s inhibition of NLRP3 in rat neuron necroptosis. (**A**) Flowchart outlining the experimental procedure at the cellular level; (**B**) Western blot analysis was conducted to quantify NLRP3 inflammasome protein expression, *n* = 4; (**C**) ELISA was used to measure IL-1β concentrations in culture media, *n* = 4;(**D**) ROS levels were assessed by DHE staining after fixation with 4% paraformaldehyde, observed under a laser scanning confocal microscope at 200x magnification, *n* = 6, (**E**) LDH levels in culture media were assayed, *n* = 6. (**F**) Experimental flowchart: male rats were subjected to lentiviral-mediated RNA interference, followed by tBHQ/QVD administration and MCAO; (**G**) Western blot analysis was performed to evaluate protein expression levels of PARK7, Nrf2, NQO1, and HO-1, *n* = 4; (**H**) NLRP3 inflammasome protein expression was also analyzed in ischemic brain tissue by Western blot, *n* = 4. ^*^p < 0.05

After studying the regulation of PARK7 on the Nrf2/ARE signaling pathway, this experiment further investigated whether the downstream NLRP3 inflammasome- mediated inflammatory response was also affected. The cellular experimental procedure is illustrated in Figure 5A: after lentivirus-mediated PARK7 knockdown and 12 days of incubation, neurons were pretreated with QVD (5 μM) and tBHQ (50 μM) for 30 min, followed by OGD for 2 h and reperfusion for 12 h. Cellular results indicated that in the sh PARK7 group, the inhibition of NLRP3 inflammasome was weakened, with increased expression levels of NLRP3 inflammasome and its activated forms, Cle-caspase1, and Cle-IL-1β proteins (Fig. 5BC), accompanied by corresponding increases in ROS and LDH levels (Fig. 5DE). The addition of tBHQ to the knockdown group resulted in enhanced activation and nuclear translocation of Nrf2, demonstrating statistically significant differences relative to the PARK7 knockdown group (Fig. 4CI). Simultaneously, the NLRP3 inflammasome’s constituent and activated proteins were downregulated, showing statistically significant differences compared to the OGD+QVD group (Fig. 5BC), and both ROS and LDH were significantly decreased compared to the sh PARK7 group (Fig. 5DE).

After clarifying the regulatory effect of PARK7 on the NLRP3 inflammasome-mediated inflammatory response at the cellular level, we further verified it in vivo, with the rat experimental flowchart shown in Fig. 5F: following lentiviral transfection, tBHQ (20 mg/kg, i.p.) was administered at 30 min pre-ischemia, 6 h later, and pre-reperfusion, while QVD (1.5 mg/kg, i.p.) was given 30 min pre-ischemia and at reperfusion onset, after which rats underwent MCAO (2 h ischemia/24 h reperfusion). In vivo results indicated that PARK7 knockdown downregulated Nrf2, NQO1, and HO-1, and intraperitoneal tBHQ injection promoted Nrf2 nuclear translocation; Western blot analysis showed that compared with the shPARK7 group, the tBHQ+shPARK7 group had significantly increased Nrf2 protein levels and downregulated NLRP3 inflammasome and Cle-IL-1β proteins (Fig. 5GH). Thus, tBHQ can reverse the negative regulatory effect of PARK7 on the NLRP3 inflammasome during ischemia/hypoxia-induced necroptosis in rat cortical neurons, though the specific underlying mechanism needs further exploration.

### PARK7 regulates Nrf2-Keap1 to downregulate NLRP3 inflammasome- mediated inflammatory responses in neuronal necroptosis

**Fig. 6:**
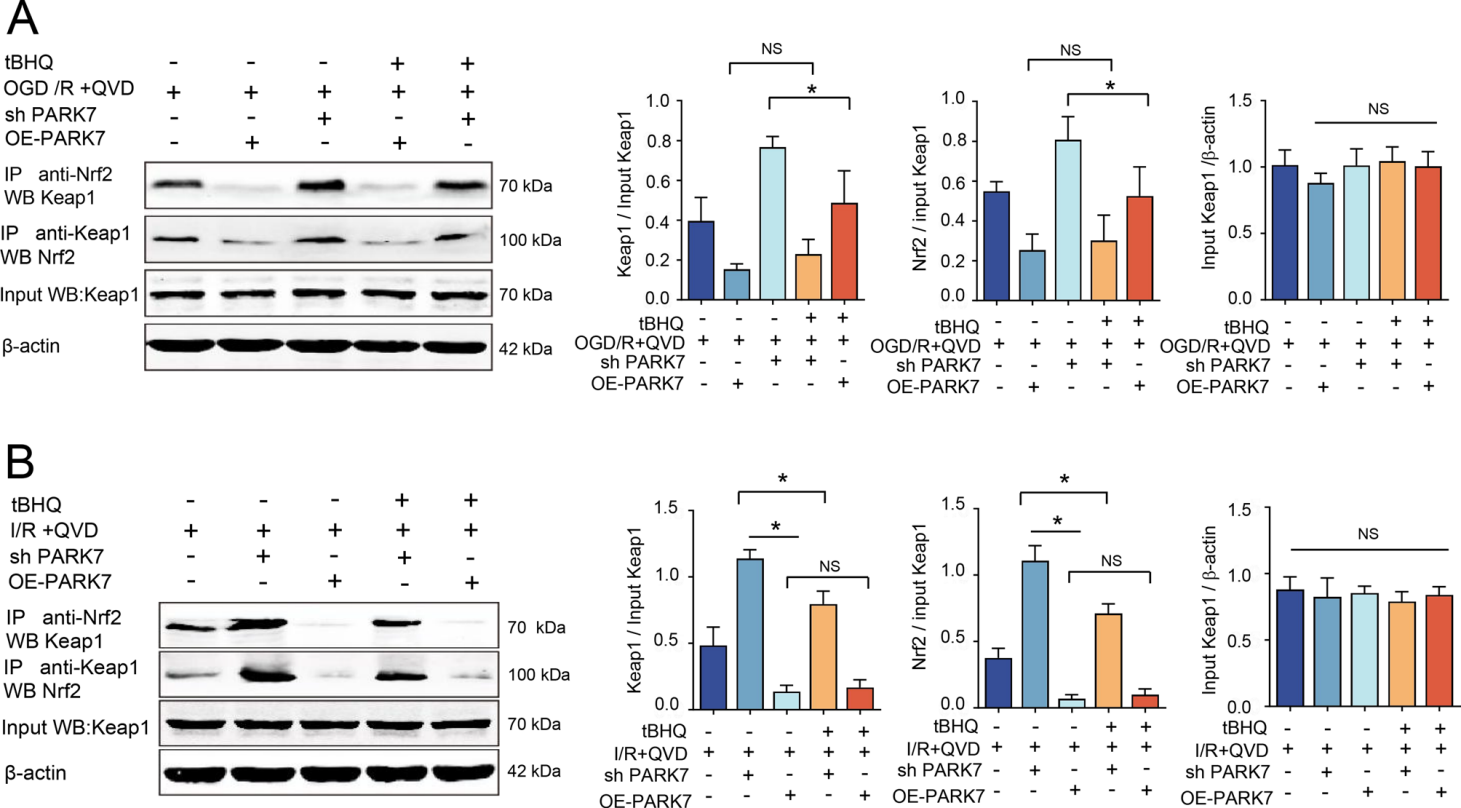
PARK7 controls NLRP3 inflammasome-mediated inflammation in neuronal necroptosis by regulating the Nrf2-Keap1 site. (**A**) Co-IP analysis of the Nrf2-Keap1 interaction in cultured cells. Lysates were immunoprecipitated with anti-Nrf2 or anti-Keap1, and the bound proteins were analyzed by immunoblotting with the corresponding antibody, *n* = 4; (**B**) Co-IP analysis of Nrf2-Keap1 binding in mouse brain tissue.Lysates from treated groups were processed as in (**A**). Input controls verify uniform protein loading, *n* = 4. ^*^p < 0.05

Under normal conditions, Nrf2 binds to its inhibitory protein Keap1 and exists in the cytoplasm. Upon stimulation by oxidative stress, Nrf2 is activated, dissociates from Keap1, and enters the nucleus [20, 21]. In vitro studies revealed that PARK7 knockdown via lentivirus significantly enhanced Nrf2-Keap1 binding compared to the OGD+QVD group, while PARK7 overexpression substantially attenuated this protein interaction relative to controls. After lentiviral interference with PARK7 and subsequent activation of Nrf2 with tBHQ, immunoprecipitation results showed that the amount of Nrf2 bound to Keap1 in the sh PARK7+tBHQ group was significantly decreased compared to the sh PARK7 group (Fig. 6A), and Nrf2 nuclear translocation was increased (Fig. 4C). The binding affinity of Nrf2 to Keap1 showed no statistically significant variation between the OE-PARK7 + tBHQ group and the OE-PARK7 group (Fig. 6A).

Vivo experiments conducted on rats, after lentiviral interference with PARK7, QVD intraperitoneal injection was used to induce necroptosis. Impaired cortical brain tissue from rats after middle cerebral artery ischemia injury was subjected to immunoprecipitation. PARK7 overexpression significantly attenuated Nrf2-Keap1 binding, an effect that remained unaltered following subsequent tBHQ administration (Fig. 6B). In the PARK7 knockdown group, Nrf2 binding to Keap1 increased, upon introduction of tBHQ treatment, the binding amount between the two significantly decreased (Fig. 6B). The above immunoprecipitation results support that PARK7 negatively regulates the binding of Nrf2 to Keap1 in necroptosis. Therefore, PARK7 directly acts on Nrf2, inhibiting its binding to Keap1 in necroptosis, thereby promoting Nrf2 nuclear translocation without affecting the total protein expression level of Keap1 (Fig. 6 AB).

### PARK7 regulates necroptosis signaling pathway by interfering with Nrf2 protein stability

**Fig. 7:**
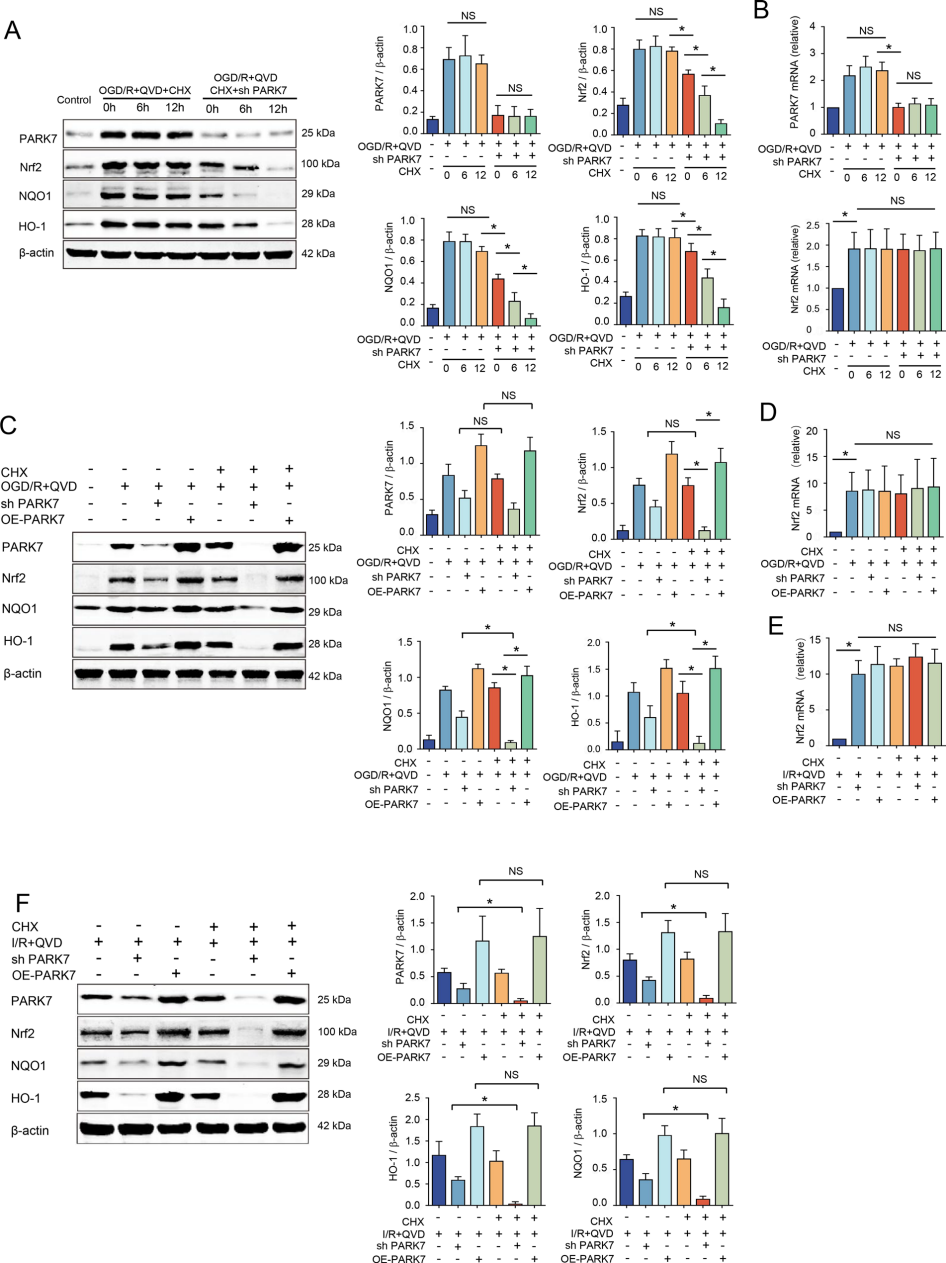
PARK7 controls necroptosis pathway by disrupting Nrf2 protein stability. (**A**) Neurons transfected with lentiviral shPARK7 were cultured for 12 days, pretreated with CHX (50 μM) at 0, 6, and 12 h, followed by QVD (5 μM; 30 min) and OGD 2 h/R 12 h. Western blot analysis assessed PARK7, Nrf2, NQO1, and HO-1 levels, *n* = 4; (**B**) RT-qPCR was conducted to assess the mRNA levels of PARK7 and Nrf2, *n* = 4; (**C**) Lentiviral knockdown and overexpression of PARK7, followed by CHX treatment, and Western blot was used to detect the protein levels of PARK7, Nrf2, NQO1, and HO-1, *n* = 4; (**D**) RT-qPCR was performed to detect Nrf2 mRNA levels, *n* = 4. (**E**) CHX (3 mg/kg) and QVD (1.5 mg/kg) were intraperitoneally administered 6 hours and 30 minutes before ischemia, respectively. QVD was additionally injected at the onset of reperfusion. RT-qPCR was conducted to detect Nrf2 mRNA levels, *n* = 4; (**F**) Western blot analysis was used to assess the protein levels of PARK7, Nrf2, NQO1, and HO-1, *n* = 4. ^*^p < 0.05; NS, not significant

Cycloheximide (CHX), a protein translation inhibitor, specifically targets peptide chain elongation [22]. In vitro study, CHX was applied at a concentration of 50 μM and grouped according to pre-treatment durations of 0 h, 6 h, and 12 h.After OGD/R + QVD treatment, the expression levels of PARK7, Nrf2, NQO1, and HO-1, both at the protein and RNA levels, remained unchanged in all groups without statistical significance. Upon lentiviral knockdown of PARK7, with prolonged CHX exposure (0 h, 6 h, 12 h), the protein levels of Nrf2, NQO1, and HO-1 gradually decreased, with the lowest protein levels observed in the sh PARK7 + CHX 12 h group (Fig. 7A). RT-qPCR analysis of PARK7 and Nrf2 expression revealed no significant differences in mRNA levels among the three varying CHX exposure duration groups of OGD/R+QVD+CHX and OGD/R+QVD+CHX+sh PARK7 (Fig. 7B), prompting the selection of a 12 h CHX exposure duration for subsequent experiments. After knocking down PARK7 and treating with CHX, the total protein level of Nrf2 was significantly reduced compared to the sh PARK7 group, accompanied by a marked decrease in NQO1 and HO-1 protein expression (Fig. 7C). Meanwhile, the protein expression levels of Nrf2, NQO1, and HO-1 showed no statistically significant differences when comparing the OE-PARK7 group with the OE-PARK7 + CHX group (Fig. 7C). RT-qPCR results indicated that lentiviral knockdown or overexpression of PARK7, with or without CHX addition, did not significantly alter Nrf2 mRNA levels compared to OGD/R+QVD groups (Fig. 7D).

In a rat model of MCAO-induced necroptosis, knocking down PARK7 and inhibiting protein translation with CHX resulted in no significant differences in Nrf2 mRNA levels among the experimental groups (Fig. 7E). As Nrf2 protein stability decreased, Nrf2 was gradually degraded, leading to a significant reduction in Nrf2 and its nuclear transcription proteins NQO1 and HO-1 (Fig. 7F). Conversely, in the PARK7 overexpression group, CHX treatment did not significantly alter the protein levels of Nrf2, NQO1, and HO-1 (Fig. 7F). These results, from both cellular and in vivo rat studies, demonstrate that PARK7 blocks necroptosis-induced brain tissue damage under ischemia and hypoxia by regulating Nrf2 stability.

### PARK7/Nrf2 downregulation reduces NLRP3 inflammasome in ischemic brain injury-induced necroptosis in rats

**Fig. 8:**
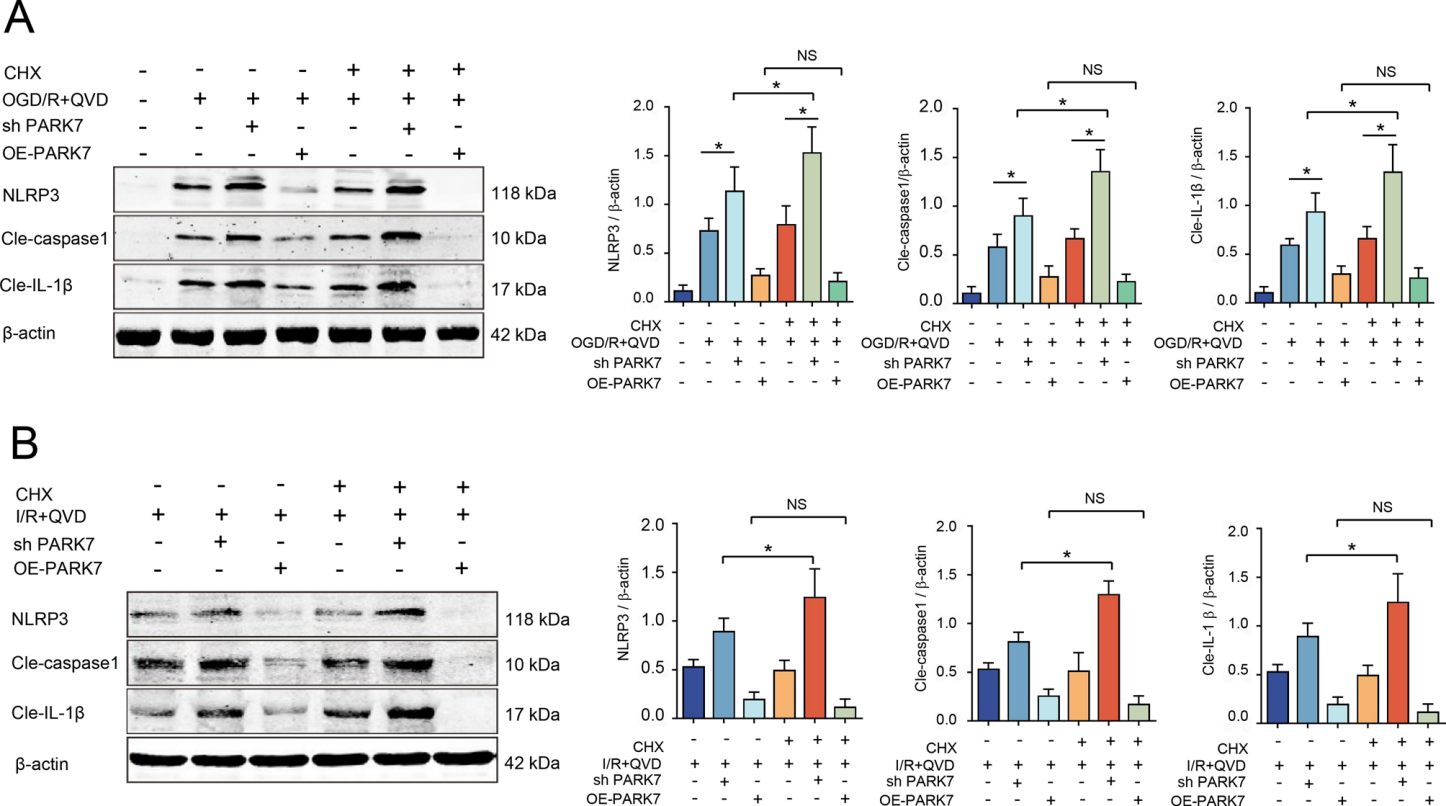
Downregulation of PARK7/Nrf2 reduces NLRP3 inflammasome levels in rat ischemic brain injury induced Necroptosis. (**A**) Following lentiviral transfection of neurons, the cells were pretreated with CHX (50 μM) for 12 hours and QVD (5 μM) for 30 minutes, followed by oxygen-glucose deprivation and reperfusion. Western blot analysis was used to detect the expression levels of NLRP3, Cle-caspase1, and Cle-IL-1β, *n* = 4; (**B**) in a rat model of MCAO to induce necroptotic injury, CHX (3 mg/kg) was administered intraperitoneally 6 hours before ischemia. Western blot analysis was performed to detect the protein levels of NLRP3, Cle-caspase1, and Cle-IL-1β, *n* = 4. ^*^p < 0.05; NS, not significant

In the necroptosis signaling pathway, following lentiviral knockdown of PARK7 and protein translation inhibition with cycloheximide (CHX) in rat cortical neurons, Nrf2 stability was reduced, leading to partial degradation. To further explore the regulation of Nrf2 stability on inflammatory responses, Western blot analysis showed that in the shPARK7+CHX group, protein levels of the NLRP3 inflammasome and its activated inflammatory protein Cle-IL-1β were elevated compared to the shPARK7 group. The comparative assessment demonstrated maintained Nrf2 stability in both experimental groups (OE-PARK7 and OE-PARK7+CHX), with parallel expression patterns of NLRP3 inflammasome components showing no statistically significant variation (Fig. 8A). Further validation in a rat MCAO model showed that upon PARK7 knockdown and CHX-mediated inhibition of protein translation, levels of NLRP3 and its activated products (Cle-caspase1 and Cle-IL-1β) were significantly elevated, consistent with prior results. In the PARK7 overexpression group, NLRP3 inflammasome protein levels showed no significant changes following CHX treatment (Fig. 8B).

## Discussion

After the onset of acute cerebral infarction, failure to promptly reverse ischemia leads to the expansion of the necrotic core and subsequent neuronal death. This process is notably exacerbated by necroptosis, a highly pro-inflammatory mode of regulated cell death, resulting in the substantial release of damage-associated molecular patterns (DAMPs). These DAMPs elicit a robust local inflammatory response in the affected region, which directly contributes to neuronal loss, heightened oxidative stress, cerebral edema, blood-brain barrier disruption, neurovascular unit impairment, and microvascular dysfunction, collectively amplifying secondary brain injury [23]. Additionally, the inflammatory response associated with stroke is not confined to the ischemic core; it can propagate throughout the brain, persistently influencing the post-stroke pathological trajectory and clinical outcomes [23]. Therefore, elucidating the mechanisms underlying post-infarction inflammation may offer novel therapeutic avenues for stroke treatment. This study demonstrates that the NLRP3 inflammasome is activated within the necroptotic signaling pathway following acute ischemic brain injury, thereby driving neuroinflammation. Notably, oxidative stress—the upstream trigger—activates the inflammasome while simultaneously inducing the transcription factor Nrf2. Activated Nrf2 then upregulates the expression of antioxidant genes such as HO-1, enhancing cellular ROS scavenging capacity. Consequently, this attenuates the key signals that trigger NLRP3 inflammasome activation at its source, ultimately establishing an intrinsic negative feedback loop of “oxidative stress → Nrf2 activation → inflammation suppression.” Building upon this regulatory circuit, we further found that the oxidative stress-responsive protein PARK7 mitigates ischemic penumbra injury after acute cerebral infarction by enhancing Nrf2 stability, thereby reinforcing this negative feedback mechanism and effectively suppressing neuroinflammation.

The cellular damage within the ischemic penumbra following cerebral infarction is transient and potentially reversible. Timely pharmacological intervention within the therapeutic window, when precisely targeted, can achieve significant neuroprotective efficacy in rescuing ischemic and hypoxic neurons [24]. Although caspase-dependent apoptosis represents the predominant form of programmed cell death in numerous pathological contexts, caspase-independent mechanisms have been established as critical contributors under specific conditions such as neurodegenerative disorders and cerebral ischemia [25]. Notably, the majority of delayed neuronal death is mediated by caspase-independent pathways, encompassing processes such as necroptosis, autophagic cell death, mitochondrial permeability transition-driven necrosis, and proteasome dysfunction [25, 26]. Consequently, targeting necroptosis, a delayed cell death mechanism in ischemic brain injury, holds promise as a novel neuroprotective strategy to extend the therapeutic window. This form of regulated necrosis amplifies the release of endogenous DAMPs in vivo, thereby initiating and potentiating innate immune and inflammatory responses.

Necroptosis is a regulated form of cell death mediated by the kinase activities of RIPK1 and RIPK3. Activation of RIPK1 promotes its association with RIPK3 to form the necrosome complex [27]. This interaction then activates the kinase function of RIPK3, which phosphorylates MLKL, a key executor specific to necroptosis [5]. Upon phosphorylation, MLKL oligomerizes and translocates to cellular and organelle membranes, resulting in membrane destabilization and eventually, cell death through membrane rupture [28]. In this study, we used QVD—a pan-caspase inhibitor—to suppress apoptotic signaling, thereby inducing neuronal necroptosis through oxygen-glucose deprivation. We further validated necroptotic activation by detecting elevated expression of RIP1, RIP3, and MLKL, whereas the addition of Nec-1 suppressed their expression, thereby confirming the occurrence of necroptosis in this model. Furthermore, prolonged reoxygenation aggravated neuronal injury, accompanied by upregulated NLRP3 inflammasome expression as shown by Western blot and immunofluorescence analyses, indicating an enhanced inflammasome-mediated inflammatory response.

The NLRP3 inflammasome stands out as one of the most comprehensively studied inflammasomes. When inflammatory signals related to pathogens and tissue damage are present, NLRP3 inflammasomes can be activated and serve as a signaling platform in the inflammatory response [29]. Following apoptosis or necrosis, damaged cells release a large number of uncleared active substances or damage-associated molecular patterns (DAMPs). Studies have shown that even non-apoptotic or non-necrotic damaged cells express high levels of DAMPs on their surface [30]. Therefore, in acute ischemic cerebrovascular diseases, cell death leads to the production of damage-associated molecular patterns (DAMPs). These released DAMPs (such as ATP, mtDNA, ROS, etc.) act as critical priming signals by activating pattern recognition receptors (e.g., TLR4) and the NF-κB pathway, upregulating the expression of NLRP3 and pro-inflammatory cytokines such as IL-1β, thereby priming the inflammasome for activation [30]. More importantly, DAMPs also serve as potent activation signals, directly triggering the assembly and activation of the NLRP3 inflammasome through multiple synergistic mechanisms, such as: K^+^ and Ca^2 +^ ion channel dysregulation, mitochondrial dysfunction, lysosomal rupture, and endoplasmic reticulum stress [31]. In the central nervous system, the NLRP3 inflammasome is mainly located in microglial cells but can also be found in astrocytes and neurons [31]. However, research on the inflammatory response in neurons remains limited. In 2013, a study by Fann DY et al. demonstrated that primary cortical neurons subjected to glucose deprivation, hypoxia, or simulated ischemia/reperfusion displayed elevated levels of NLRP1 and NLRP3 inflammasome components, along with their associated cytokines, IL-1β and IL-18. The research further revealed that intravenous immunoglobulin (IVIg) treatment protects neurons in stroke models by suppressing the activation of NLRP1 and NLRP3 inflammasomes [32]. In 2023, the research group further elucidated that the NF-κB and MAPK signaling pathways play a critical role in modulating the expression and activation of NLRP1 and NLRP3 inflammasomes in primary cortical neurons and brain tissue under ischemic conditions [33]. Treatment with IVIg significantly inhibited the activation of NF-κB and MAPK signaling pathways in primary cortical neurons under ischemic conditions, leading to a marked reduction in the expression and functional activity of NLRP1 and NLRP3 inflammasomes [34]. As neurons serve as the primary structural and functional components of the nervous system, elucidating the mechanisms underlying their injury could accelerate the development of precise therapeutic interventions for patients with cerebral infarction.

PARK7, also known as DJ-1, is most commonly associated with autosomal recessive hereditary Parkinson’s disease. It was first identified as an oncogene product in 1997 and named accordingly. PARK7 is a conserved protein made up of 189 amino acids and is a member of the Thi/PfpI protein superfamily of molecular chaperones [35]. In brain cells, PARK7 is primarily distributed in the cytoplasm and membrane gaps of neurons and glial cells, with smaller amounts found in the nucleus and mitochondria [36]. When cells are exposed to oxidative stress, PARK7 translocates to the mitochondria, and with continued stimulation, it may relocate to the nucleus, providing neuroprotective effects [36]. Oxidative stress-induced damage from ischemia/reperfusion is a major secondary injury mechanism in the cell death signaling pathway of acute ischemic stroke. PARK7 is pivotal in modulating the oxidative stress response during stroke, primarily through its antioxidant properties, which are mediated by the sensitivity of its Cysteine 106 (Cys106) residue to reactive oxygen species (ROS) [37]. Studies have shown that in human neuroblastoma and rat primary neurons, PARK7 integrates mitochondrial-related pathways and plays an essential role during the early stages of stroke [38]. Mitochondria are pivotal in scavenging free radicals, and the movement of PARK7 to mitochondria aids in alleviating mitochondrial damage and decreasing the generation of mitochondrial reactive oxygen species (ROS). Furthermore, PARK7 exerts its protective effects through multiple mechanisms: (1) upregulating the expression of mitochondrial uncoupling proteins to decrease ROS generation, (2) stabilizing the interaction between PARK7 and mitochondrial Bcl-xL, and (3) preventing cytochrome c release from mitochondria, thereby inhibiting apoptotic pathways [39]. It has also been found that in PARK7-deficient co-cultures, antioxidants are unable to counteract hypoxia-induced neuronal apoptosis [40]. The antioxidant effects of PARK7 help alleviate neuronal damage and promote functional recovery, but its specific role and mechanisms in acute cerebral infarction still require further investigation.

Nrf2, a member of the leucine zipper transcription factor family, serves as a master regulator of cellular antioxidant responses. Under conditions of oxidative stress or in the presence of electrophilic agents, Nrf2 is activated and translocates to the nucleus, where it binds to the GCTGAGTCA sequence within the antioxidant response element (ARE) [41]. This interaction triggers the expression of various antioxidant genes, such as glutathione transferase, NADPH quinone reductase, γ-glutamylcysteine synthetase, and heme oxygenase 1, among others [42]. Under physiological conditions, in the absence of oxidative stress, Nrf2 is sequestered in the cytoplasm by its inhibitory partner, Kelch-like ECH-associated protein 1 (Keap1), which prevents its activation and subsequent translocation to the nucleus. During this process, Keap1 mediates the ubiquitination and degradation of Nrf2, maintaining its normal levels and thereby suppressing the expression of downstream genes [42]. tBHQ can facilitate the nuclear translocation of Nrf2, which activates the Nrf2 protein, strengthens its binding to ARE, and regulates the expression of antioxidant proteins [43]. In this study, tBHQ demonstrated a protective effect in the rat cortical neuron model of ischemia/reperfusion-induced Necroptosis, as well as in the rat middle cerebral artery ischemia-reperfusion injury model. tBHQ facilitated Nrf2 nuclear translocation, which regulated nuclear transcription factors HO-1 and NQO1, further alleviating oxidative stress damage caused by ischemia/reperfusion.

Numerous studies have demonstrated that PARK7 participates in antioxidant stress responses via its effects on Nrf2. In this experiment, we further investigated whether PARK7, within the necroptosis signaling pathway induced by ischemia-reperfusion, modulates the NLRP3 inflammasome-mediated inflammatory cascade through Nrf2. Both cellular and in vivo rat models showed increased Nrf2 nuclear translocation following hypoxia-reoxygenation stimulation. PARK7 was shown to regulate Nrf2 nuclear translocation, as knockdown of PARK7 did not affect Nrf2 gene expression but reduced Nrf2 nuclear translocation, lowered ROS levels, and inhibited the NLRP3 inflammasome-mediated inflammatory response, thereby alleviating neuronal damage. When Nrf2 was activated with tBHQ, Nrf2 nuclear translocation was increased in the PARK7 knockdown group, and neuronal and brain tissue damage was reduced in rats. Thus, under oxidative stress induced by ischemia-reperfusion, Nrf2 acts as an effector protein through which PARK7 modulates necroptosis. PARK7 functions as both an oxidative stress sensor and antioxidant agent. Our findings demonstrate that in the necroptosis pathway, PARK7 acts on Nrf2/ARE to promote the transcriptional expression of antioxidant proteins NQO1 and HO-1, thereby regulating oxidative stress and inhibiting the NLRP3 inflammasome-mediated inflammatory response in neurons and brain tissues under ischemia-reperfusion necroptosis. However, the exact mechanism by which PARK7 activates Nrf2 requires further exploration. In subsequent experiments, we will delve deeper into whether PARK7 directly activates Nrf2 in the necroptosis pathway or achieves Nrf2 activation by releasing it from its inhibitory state.

Keap1 is a critical regulatory protein in cellular signaling pathways that mediates antioxidant stress responses, with its most common target being the nuclear transcription factor Nrf2 [44]. The Keap1-Nrf2/ARE signaling pathway plays a crucial role in the body’s antioxidant, anti-inflammatory, and anticancer pathways. Among the many downstream target proteins of Nrf2, NQO1 and HO-1 are the most studied and have gained significant attention as antioxidant proteins [45]. In this experiment, we found that in the Necroptosis signaling pathway, following ischemia-reperfusion injury, the expression of Nrf2 increased, and the levels of NQO1 and HO-1 correspondingly elevated. This increase was associated with the downregulation of ROS, thereby inhibiting oxidative stress responses. When PARK7 was downregulated, there was a reduction in Nrf2 nuclear translocation, leading to decreased levels of NQO1 and HO-1. Conversely, when PARK7 was upregulated, there was an increase in Nrf2 nuclear translocation and a corresponding rise in NQO1 and HO-1 levels. This enhanced Nrf2 activity exerted a negative regulatory effect on the NLRP3 inflammasome-mediated inflammatory response. Thus, the Keap1-Nrf2/ARE pathway is involved in the inflammatory response within the Necroptosis signaling pathway, and PARK7 can protect cells from inflammation-related damage by regulating the Keap1-Nrf2/ARE signaling axis. Nrf2 inhibition is not only associated with the coupling state of Keap1 but also with the direct degradation of Nrf2. Therefore, further investigation into the regulatory mechanism of PARK7 on Nrf2 is warranted.

PARK7 is a multifunctional protein that serves as a redox sensor and an effector in several cellular protective pathways. PARK7 is capable of regulating multiple transcription factors, including the nuclear factor Nrf2, phosphoinositide 3-kinase (PI3K)/protein kinase B (PKB), and p53 signaling pathways, although its role in regulating the inflammatory response remains underexplored [46]. Dimethyl fumarate (DMF) and monomethyl fumarate (MMF) have physiological effects similar to those of PARK7. Studies have demonstrated that DMF/MMF can prevent the binding of Nrf2 to its inhibitor, Keap1 permits Nrf2 to move into the nucleus, initiating cellular protection and antioxidant cascades [47]. The Nrf2-Keap1 inhibitory complex is the Nrf2-dependent drug target of DMF/MMF. In primary human alveolar type 2 cells, DMF/MMF has been shown to act on the Nrf2-Keap1 complex, serving as an anti-inflammatory activator of Nrf2 [48]. DMF/MMF and PARK7 may exist together under similar pathophysiological conditions.The dynamic balance of the Nrf2-Keap1 axis might regulate the involvement of both DMF/MMF and PARK7 in the Nrf2 signaling cascade in a complementary manner [45]. Specific activation of Nrf2 by DMF/MMF can directly upregulate genes such as NQO1, and the expression of NQO1 increases in tandem with the cascading effects of PARK7 [49]. Additionally, the expression trends of HO-1 and the glutamate system are consistent with those of Nrf2. Both DMF/MMF and PARK7 show similar expression patterns in various oxidative stress and inflammation models [48]. However, as an upstream activator of the Nrf2 cascade, the interaction of PARK7 in the inflammatory response mechanisms remains inadequately studied.

Through immunoprecipitation, we found that PARK7 negatively regulates the binding of Nrf2 to Keap1. In the experimental group with PARK7 knockdown, the addition of tBHQ reduced the binding between Nrf2 and Keap1, leading to an increase in Nrf2 activation. This suggests that the antioxidant mechanisms of PARK7 and tBHQ are similar, both promoting the deubiquitination of Nrf2, causing its dissociation from Keap1, and thereby activating Nrf2 and promoting its nuclear translocation. To explore whether PARK7 interferes with Nrf2 through other mechanisms, we continued our research and found that there were no significant changes in Nrf2 gene expression levels before and after lentiviral-mediated PARK7 knockdown. When CHX was used to inhibit protein translation, we observed a decrease in Nrf2 protein levels in the PARK7 knockdown group, whereas no notable alterations were seen in the overexpression group. These results suggest that PARK7 functions by facilitating the dissociation of Nrf2 from Keap1, impeding Keap1-induced ubiquitination and degradation of Nrf2. Consequently, this enhances the stability of Nrf2, resulting in its activation and nuclear translocation.

## Conclusions

In the treatment of acute cerebral infarction, extending the therapeutic window does not merely aim to shorten the time to vessel recanalization, but rather focuses on developing neuroprotective strategies capable of effectively intervening in the progressively evolving injury mechanisms during ischemia and reperfusion. Necroptosis constitutes a regulated and persistently active cell death program that is triggered secondary to early ischemic events. Consequently, therapeutic interventions targeting this pathway may present a broader temporal opportunity. This form of cell death ultimately culminates in plasma membrane rupture and incites an inflammatory response. Historically, research on neuroinflammatory mechanisms has predominantly centered on glial cells, while the active involvement of neurons in this process and their underlying regulatory mechanisms remain insufficiently elucidated. Notably, as the primary functional unit of the nervous system, the survival and synaptic integrity of neurons fundamentally determine the preservation of neural circuitry and the clinical prognosis of patients.

**Fig. 9:**
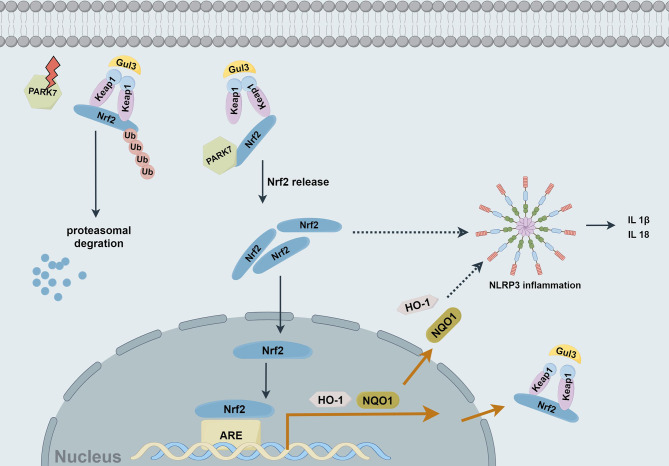
A signaling pathway diagram of PARK7-regulated NLRP3 inflammasome pathway in ischemic neuronal necroptosis. After binding to Nrf2, PARK7 can dissociate it from Keap1, preventing ubiquitination and degradation, thereby enhancing the stability of Nrf2 and promoting its nuclear translocation. This initiates the Nrf2/ARE signaling pathway, upregulates the levels of NQO1 and HO-1, enhances antioxidant and anti-inflammatory effects, and downregulates the inflammatory responses mediated by the NLRP3 inflammasome in the signaling pathway of ischemic hypoxia-induced neuronal necroptosis

By transcending the traditional research paradigm confined by cell-type classification, this study precisely directs the interventional strategy toward the final functional effector of neurological recovery—the neuron itself. This approach not only deepens the mechanistic understanding of neuroinflammation, but also offers a novel theoretical foundation and a potential target-selection pathway for developing functionally oriented neuroprotective therapies. This research reveals that following the onset of necroptosis, cells activate the NLRP3 inflammasome to proactively initiate inflammatory signaling. Concurrently, intracellular oxidative stress upregulates the expression of PARK7 and the transcription factor Nrf2, thereby establishing an endogenous defensive axis that downregulates the inflammatory response (Fig. 9). Consequently, targeting this axis not only mitigates neuron-driven inflammation within the necroptosis signaling cascade but also, when combined with vessel recanalization therapy, holds promise for alleviating reperfusion injury, suppressing secondary inflammatory cascades, and preserving the structural and functional integrity of the neurovascular unit, thereby breaking the constraints imposed by the traditional narrow time window, offering effective neuroprotection to a greater number of ischemic stroke patients, and improving their long-term prognosis. This represents a significant evolution in stroke treatment strategy—shifting from the paradigm of “racing against time for recanalization” to one that “emphasizes both recanalization and post-recanalization protection.”

## Data Availability

Not applicable.
